# Cognitive reserve, neuropathology, and progression towards Alzheimer’s disease

**DOI:** 10.18632/aging.204909

**Published:** 2023-07-14

**Authors:** Monica E. Nelson, Ross Andel, Jakub Hort

**Affiliations:** 1 School of Aging Studies, University of South Florida, Tampa, FL 33612, USA; 2 Edson College of Nursing and Health Innovation, Arizona State University, Phoenix, AZ 85004, USA; 3 Department of Neurology, Second Faculty of Medicine, Charles University and Motol University Hospital, Prague, Czech Republic

**Keywords:** dementia, neuropathology, Alzheimer’s disease, MRI, volumetry

With prevalence of dementia expected to almost triple over the next 30 years [1], there has been much research effort aimed at disentangling the complicated three-way relationship between contextual factors associated with Alzheimer’s disease (AD), biological manifestations of AD, and its clinical phenotype in hopes of finding effective targets for prevention or intervention. Research has frequently used the cognitive reserve concept [2], which attempts to capture individual differences stemming from genetic and early- or mid-life contextual factors, to explain what allows some people to maintain cognitive abilities and postpone progression to dementia in the presence of neuropathology substantial enough to suggest otherwise. What contributes to this “resilience” [3], that is why some successfully cope with progressive neuropathology while others cannot tolerate the same level of neurodegeneration, is not fully understood.

One major limitation of this research is that studies often base inferences about variability in cognitive or clinical outcomes as a function of a certain marker of reserve without actually measuring neuropathology [2], thus presenting an incomplete view of the three-way associations between cognitive reserve, neuropathology, and cognitive/clinical outcomes. This approach arguably limits progress in understanding mechanisms underlying resilience presumably attributable to cognitive reserve [3]. More recently, research groups have been considering these three necessary components simultaneously to assess these relationships, whether cross-sectionally or longitudinally.

**Figure 1:**
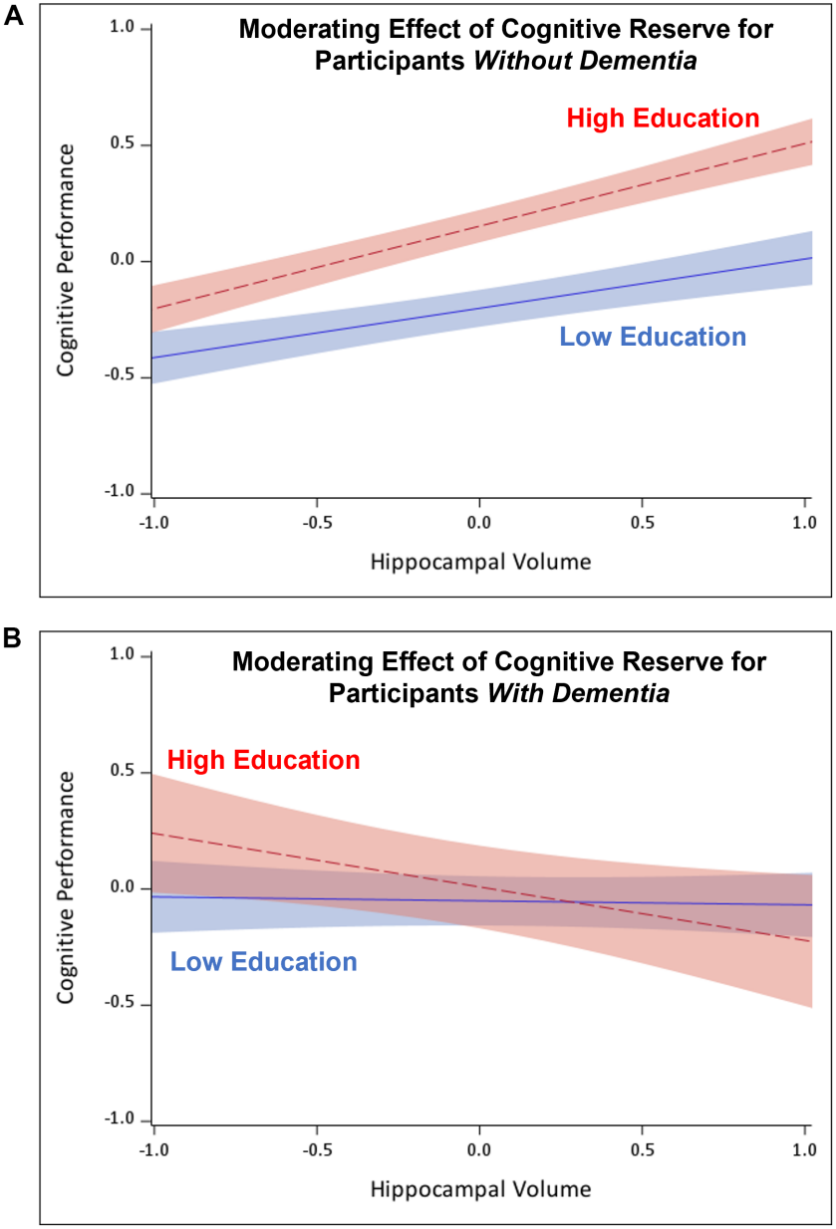
**Moderating effect of cognitive reserve on the brain volume-cognition relationship.** *Note.* Cognitive performance and hippocampal volume are presented in z-scores (i.e., mean = 0, *SD* = 1). (**A**) Indicates a stronger positive relationship between brain volume and cognitive performance for participants without dementia who have high cognitive reserve. (**B**) Indicates a strong negative relationship between brain volume and cognitive performance for participants with dementia who have high cognitive reserve.

For example, we recently assessed the moderating effect of cognitive reserve proxy variables on the relationship between hippocampal or total gray matter volume and several domains of cognition in participants without and with dementia syndrome [4]. Using cross-sectional data from the Czech Brain Aging Study, a longitudinal cohort study from two memory clinics in the Czech Republic [5], we assessed the inter-link between cognitive reserve, neuropathology, and cognitive functioning among participants with subjective cognitive decline, mild cognitive impairment, and dementia. We found that, among non-demented older adults who had *high cognitive reserve*, in this case favorable scores on two contextual factors representing cognitive reserve (i.e., years of education, occupational position), the *positive* association between brain volume and cognition was stronger than among those with low cognitive reserve. In older adults with dementia, this pattern was reversed –a stronger *positive* relationship between brain volume and cognitive outcomes emerged among participants with *low cognitive reserve*, whereas participants with *high cognitive reserve* had a stronger *negative* relationship between brain volume and cognitive outcomes. Figure 1 presents these divergent patterns in participants without and with dementia.

Taking a bird’s eye view at this area of research, progress towards understanding the inter-relationship between contextual factors representing cognitive reserve, neuropathology, and cognitive outcomes relies on addressing the following. First, it is important to assess older adults across the cognitive spectrum within the same study when possible so that the shape of the observed relationships across levels of cognitive impairment can be juxtaposed immediately. Specifically, at what point is cognitive reserve protective of brain health and compensatory against neuropathology through resilience vs. where on the progression across levels of cognitive impairment does it lose its effectiveness? Some, including our work [4], have suggested that this relationship may be U-shaped [6] which helps explain mixed findings in different studies.

Second, a range of AD biomarkers, including beta-amyloid and tau (measured via cerebrospinal fluid or positron emission tomography) and neurodegeneration (measured via structural magnetic resonance imaging), should be investigated synergistically but also individually to determine whether the use of a spectrum of AD biomarkers may be influencing associations found among cognitive reserve, brain health, and cognition. Since the development of underlying AD pathology is proposed to occur in stages across a cascade that includes a beta-amyloid buildup, accumulation of tau protein, and subsequent neurodegeneration reflected in atrophy across important brain regions [7], it is possible that studies including different groups of AD biomarkers may observe different results regarding how cognitive reserve moderates associations between brain health and cognition simply as a function of what biomarkers were measured. These challenges are addressed by the ATN AD diagnostic criteria [7]. For example, studies focused on healthy older adults with beta-amyloid positivity may indicate a compensatory role for cognitive reserve whereas including markers of brain volume/neurodegeneration may suggest a protective role of cognitive reserve since abnormal beta-amyloid reflects a more upstream neuropathological process than neurodegeneration.

Third, longitudinal studies are needed to track how cognitive reserve operates in individuals as they progress across the whole range of the cognitive spectrum from normal to AD dementia. Since cross-sectional studies only represent a snapshot of these associations, it is possible that mean differences among participants may not reflect prior disease-related changes in AD neuropathology and may, therefore, present a distorted view of the linkages between cognitive reserve indicators, neuropathology, and cognitive outcomes and the neuropathology-to-clinical impairment sequence. Additionally, there may be developmental differences between participants that influence these associations but cannot be accounted for in cross-sectional studies. By assessing these three key variables (cognitive reserve indicators, neuropathology, and cognitive outcomes) over time, a more nuanced understanding of what factors may lead to better brain health or coping with neuropathology can be obtained.

Finally, knowledge in this area comes almost exclusively from findings from university-based, relatively sociodemographically homogeneous samples [8]. Even though our study represents one of the first to come from Eastern Europe [4], future work should be conducted in additional populations, representing geographic, racial, and socioeconomic diversity. By assessing cognitive reserve in distinct populations, a more complete understanding of how cognitive reserve relates to neuropathology and cognition and whether these associations may be affected by distinct macro-level contextual differences among populations can be established. Disentangling these complex relationships may provide a critical step in reducing the impact of dementia on society.
